# Localized gastric amyloidosis successfully treated with endoscopic submucosal dissection: A case report

**DOI:** 10.1097/MD.0000000000028422

**Published:** 2021-12-23

**Authors:** Gyu Man Oh, Seun Ja Park, Jae Hyun Kim, Kyoungwon Jung, Sung Eun Kim, Won Moon, Moo In Park, Hee-Kyung Chang

**Affiliations:** a Department of Internal Medicine, Kosin University College of Medicine, Busan, Korea; b Department of Pathology, Kosin University College of Medicine, Busan, Korea.

**Keywords:** amyloidosis, case report, endoscopic submucosal dissection, endoscopic ultrasonography

## Abstract

**Rationale:**

Amyloidosis is a general term that refers to the extracellular deposition of amyloid. The amyloid can also be deposited in a single organ. However, cases of localized gastric amyloidosis have rarely been reported. Here, we report a case of localized gastric amyloidosis that was successfully treated with endoscopic submucosal dissection.

**Patient concern:**

A 60-years-old man underwent esophagogastroduodenoscopy as part of a regular check-up without any comorbidities or symptoms.

**Diagnostics:**

A 12 mm-sized, round, elevated lesion with a central depression, which was covered with normal mucosa, and located on the greater curvature of the lower body of the stomach was discovered during endoscopy. Subsequently, endoscopic ultrasonography was performed, which revealed a 11.7 mm-sized, hypoechoic, heterogeneous lesion located in the muscularis mucosa and submucosa. A biopsy was performed, and amyloid deposition was confirmed. Although other investigations for checking systemic amyloidosis were performed, there were no specific findings. Therefore, the final diagnosis was localized gastric amyloidosis.

**Interventions:**

Endoscopic submucosal dissection was performed according to the patient's request and the lesion was completely removed.

**Outcomes:**

The patient was followed-up for 3 years without any recurrence.

**Conclusions:**

Endoscopic submucosal dissection can be good diagnostic and treatment option for localized gastric amyloidosis.

## 1 Introduction

Amyloidosis is a general term that refers to the extracellular deposition of amyloid, which is composed of various subunit proteins and is characterized by apple green birefringence with polarized light microscopy on Congo red staining.^[1]^ Amyloidosis is an uncommon disease, and it has several major types. Amyloidosis is divided according to the histologic type of the constituting protein into the following types: AA type (related to hematologic diseases), AL type (related to chronic inflammatory diseases), and dialysis-related. Amyloidosis can be classified into two groups such as primary or secondary, and as generalized or localized. In most cases, amyloidosis is associated with systemic diseases, such as multiple myeloma, Waldenstrom macroglobulinemia, rheumatoid arthritis, and spondyloarthropathy.

Nevertheless, amyloid deposition can be limited to a single organ.^[2]^ This means that amyloid can be accumulated locally rather than systemically. However, cases of localized gastric amyloidosis have rarely been reported. Among them, only 2 cases have been reported that were treated through endoscopic resection.^[3,4]^ Here, we report a case of localized gastric amyloidosis that was successfully treated with endoscopic submucosal dissection (ESD) and followed up for 3 years.

## 2 Case presentation

A 60-years-old man underwent esophagogastroduodenoscopy as part of a regular check-up. He had no underlying diseases or any symptoms. There were no abnormalities detected in the blood tests (complete blood count, liver profile, blood urea nitrogen, creatinine, electrolyte, and C-reactive protein), urine tests, and simple plain radiography.

**Figure 1:**
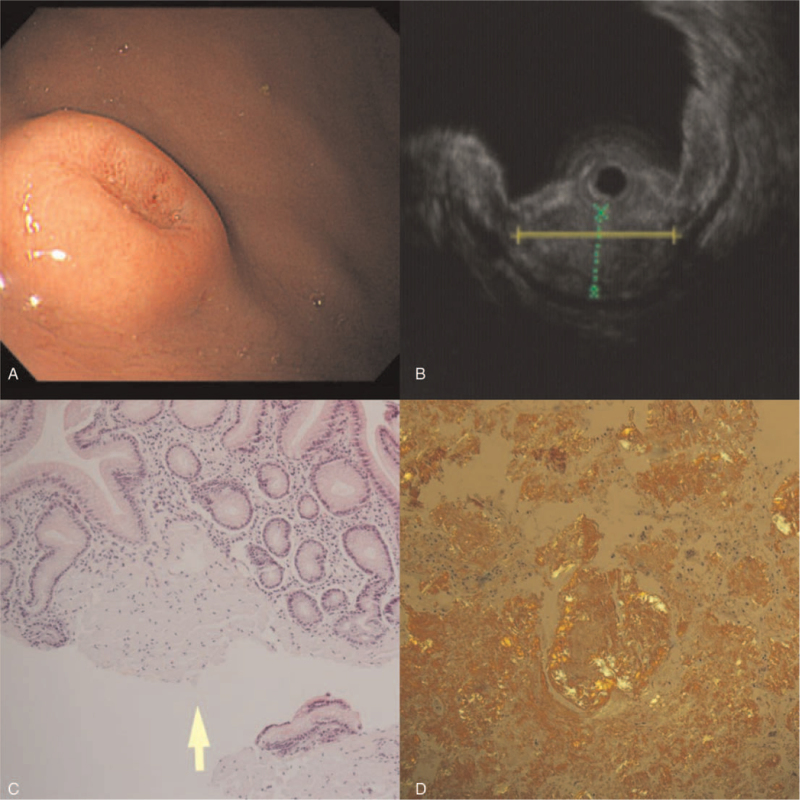
Images at initial diagnosis. White-light image (A). Endoscopic ultrasonography image (B). Hematoxylin and eosin stain (C). Congo-Red stain (D).

A 12 mm-sized, round, elevated lesion with a central depression, which was covered with normal mucosa and located on the greater curvature of the lower body of the stomach was discovered during endoscopy (Fig. 1). Endoscopic ultrasonography revealed that the lesion was 11.7 mm in size, hypoechoic, heterogeneous, and located in the second and third layers of the stomach. It was found to lie in the muscularis mucosa and submucosa. The doctor who performed the endoscopy considered the lesion to be a gastrointestinal stromal tumor and performed a surface biopsy using cold forceps.

However, under a light microscope, pink and amorphous deposits were observed in the lamina propria when stained with hematoxylin and eosin. On staining the specimen with Congo red stain followed by microscopic observation under a polarizing filter, amyloidosis was detected. Considering the possibility of gastrointestinal tract invasion in systemic amyloidosis, the patient was referred to a hematologist, rheumatologist, nephrologist, and cardiologist for a multidisciplinary approach. Chest and abdominal computed tomography; serum immunoglobulin; serum electrophoresis; tests for various autoantibodies including rheumatoid factor, anti-CCP antibody, and anti-nuclear antibody; kidney ultrasonography; spot urine protein; protein/creatinine ratio; and transthoracic echocardiography were performed. However, there were no specific findings in any of the tests. The patient was finally diagnosed with the subepithelial type of localized gastric amyloidosis and the diagnosis and prognosis were explained to the patient.

**Figure 2:**
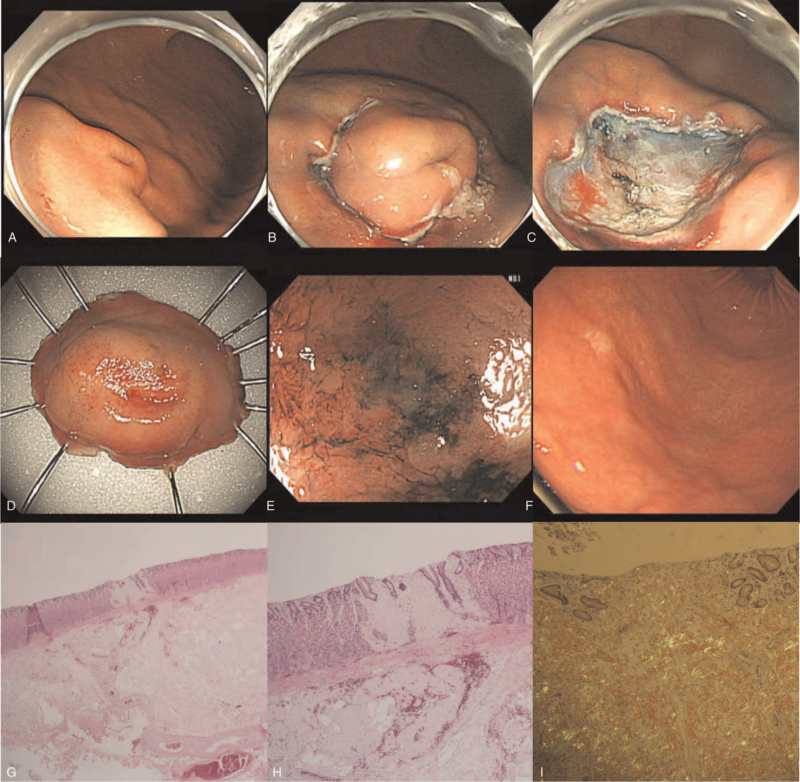
Endoscopic submucosal dissection (A, B, C). Resected tumor (D). Surface of the central ulcer in the tumor (E). Two months after the resection (F). Hematoxylin and eosin stain (G, H). Congo-Red stain (I).

Although there were no digestive symptoms or signs of bleeding from the lesion, the patient complained of anxiety and wanted the lesion to be removed. Therefore, ESD was performed according to patient's request (Fig. 2). The 8 × 2 mm submucosal tumor was removed, and the margin was cleared. The patient was followed up for 3 years without any recurrence.

## 3 Discussion and conclusions

Digestive organ involvement is relatively common in patients with amyloidosis. It is influenced by the type of amyloidosis.^[5]^ Gastrointestinal involvement was observed in 60% of AA type and 8% of AL type patients.^[6,7]^ Liver was found to be the most commonly invaded organ among the digestive organs, showing involvement in up to 90% of patients.^[8]^ Gastrointestinal amyloidosis causes symptoms such as bleeding, gastroparesis, constipation, bacterial overgrowth, and dysmotility.

AA-type amyloidosis is related to hematologic diseases such as multiple myeloma or Waldenstrom macroglobulinemia, while AL-type amyloidosis is related to systemic inflammation, such as rheumatoid arthritis and inflammatory bowel disease. If systemic amyloidosis involves the stomach, AA amyloid is mainly deposited in the lamina propria, while AL amyloid is predominantly deposited below the muscularis mucosa in general. As a result, AA amyloidosis causes an ulceration, whereas AL amyloidosis is discovered as a submucosal tumor on endoscopy.^[9]^ In this case, AL-type amyloidosis was suspected considering the endoscopic morphology. Unfortunately, direct identification of the proteins present in the amyloid deposits, whether the AA or AL type, was not performed in this case as the equipment used for analysis was unavailable at this center.

Single digestive organ deposition in amyloidosis is very rare. Twenty two cases of localized gastric amyloidosis have been reported to date.^[10]^ The endoscopic findings of gastric amyloidosis are very different depending on the type of protein and the depth of deposition. It can show thickening of the mucosal folds, increased mucosal fragility, erosion, ulcers, and submucosal tumors. Since these diverse features cannot be standardized, the endoscopy doctors may consider gastric cancer, mucosa-associated lymphoid tissue (MALT) lymphoma, erosive gastritis, nodular gastritis, Crohn's disease, sarcoidosis, and gastric polyp diseases as a differential diagnosis.

Treatment of localized gastric amyloidosis is different from that of systemic amyloidosis. In AA amyloidosis, treatment of the underlying inflammatory disease is a priority because it is a secondary amyloidosis caused by inflammation. Similarly, treatment of the underlying hematologic diseases should be considered in cases of AL amyloidosis. Kidney transplantation should be considered for patients with dialysis-related amyloidosis. Although endoscopic ultrasonography, endoscopic mucosal resection, and endoscopic submucosal resection are attractive diagnostic and treatment options for localized gastric amyloidosis (LGA), none of them has been proposed as a definitive tool.

There were 22 LGA cases reported so far, and only 2 cases were treated with ESD. This case is the 3rd case of LGA treated with ESD. Three cases including this case are compared in Table 1.^[3,4]^ In general, localized amyloidosis has a good prognosis and is known to be less likely to recur when treated.^[11]^ For this reason, many localized amyloidosis were just observed or treated symptomatically. However, if there is hematemesis, recurrent pain, or anemia in LGA patients, surgical resection is considered.^[12–14]^ Currently, ESD is attempted for treating LGA owing to the development of endoscopic surgery, but only few cases were reported because of its lower prevalence. ESD will be implemented in more LGA cases based on this case.

**Table 1:** Comparison of 3 cases of localized gastric amyloidosis treated with endoscopic submucosal dissection.

Case	Ebato et al^[3]^	Jin et al^[4]^	This case
Age	77	33	60
Sex	Female	Female	Male
Endoscopic findings	–A flat, depressive lesion –The lesion bled easily after air inflation –46 mm sized –At the greater curvature of the lower body	1) –Irregular borders and mucosa –12 mm sized –At the lesser curvature of body 2) –Regular borders and smooth surface –20 mm sized –At the fundus	–A round, elevated lesion with central depression –Covered with normal mucosa –12 mm sized –At the greater curvature side of lower body
Biopsy result	H&E staining: amyloid deposits in the mucosal and submucosal layer	H&E staining: A structureless deposition of amyloid from the submucosa to the muscle layer	Congo red stain: Apple-green birefringence in muscularis mucosa and submucosa layer under polarized light
Treatment	ESD	ESD	ESD
The reason of resection	Anemia not improved with conservative care	Worsening of epigastric pain	Patient's request
Result	Anemia improvement	No recurrence after 6 mo	Follow-up for 3 yr without recurrence

ESD = endoscopic submucosal dissection, H&E = hematoxylin and eosin.

## Author contributions

**Conceptualization:** Gyu Man Oh, Seun Ja Park.

**Data curation:** Hee-Kyung Chang.

**Resources:** Hee-Kyung Chang.

**Supervision:** Seun Ja Park.

**Writing – original draft:** Gyu Man Oh.

**Writing – review & editing:** Gyu Man Oh, Seun Ja Park, Jae Hyun Kim, Kyoungwon Jung, Sung Eun Kim, Won Moon, Moo In Park, Hee-Kyung Chang.
